# Diabetic Macular Edema: From Old Concepts to New Therapeutic Avenues

**Published:** 2015

**Authors:** Ahmad M Mansour, Jose S Pulido, J Fernando Arevalo

**Affiliations:** 1 Department of Ophthalmology, American University of Beirut, and Hariri University Hospital, Beirut, Lebanon; 2 Department of Ophthalmology, Mayo Clinic, Rochester, Minnesota (JSP); 3 Wilmer Eye Institute, The Johns Hopkins University, Baltimore, Maryland, USA (JFA)

**Keywords:** Diabetic macular edema, ultrawide field fluorescein angiography, Vascular endothelial growth factor, retinal ischemia, panretinal photocoagulation

## Abstract

Diabetic macular edema (DME) is a significant cause of blindness in the working population and is currently challenging to treat. Current interventions include focal laser or intravitreal injections. This article outlines a new treatment protocol based on the theory that peripheral ischemia is the precursor to angiogenesis, which will ultimately gather its momentum at the fovea. Extreme peripheral light laser panretinal photocoagulation (PRP) back to the equator reduces excessive production of the vascular endothelial growth factor (VEGF) in the eye. This decreases VEGF-induced DME and provides long-term protection against the development of neovascularization. Initial exacerbation of DME often accompanies PRP. Therefore, injections of anti-VEGF agents (with or without dexamethasone implants) initially can forestall worsening of DME and prevent loss of vision. However, on the other hand, applying peripheral PRP and intraocular injections can induce posterior vitreous detachment (PVD). This could help release vitreomacular adhesions (VMA) and vitreomacular traction (VMT), thereby decreasing DME severity and improving the response to intravitreal injections. In the current approach, peripheral retinal photocoagulation should stop the drive for VEGF release; moreover, laser ablation should produce secondary, accidental, and beneficial PVD. This approach precludes focal laser therapy and paves the path for prolonged intervals between anti-VEGF therapy.

## Introduction

Diabetic macular edema (DME) is a multifactorial, complex and major cause of blindness worldwide. The disease involves breakdown of the blood-retinal barriers, oxidative stress, and increased growth factors [including vascular endothelial growth factor (VEGF)] (1). Current interventions include control of systemic comorbidities (e.g., hyperglycemia, dyslipidemia, and systemic hypertension). Ophthalmic treatments include monotherapy or a combination of focal macular laser photocoagulation (argon vs. subthreshold micropulse diode) and intravitreal pharmacotherapy (triamcinolone, dexamethasone or fluocinolone implants, ranibizumab, bevacizumab, aflibercept). Several studies (2, 3) have shown that anti-VEGF therapy is somewhat effective in controlling DME, even in cases where focal macular laser treatment has failed. Following FDA approval, ranibizumab and aflibercept have become the first-choice therapy for DME (4). The introduction of ranibizumab for the management of DME has shifted the approach to this entity dramatically. Focal laser photocoagulation is still a preferred treatment for DME not involving the center of the macula (5). The Early Treatment Diabetic Retinopathy Study (ETDRS) showed that focal photocoagulation in eyes with DME reduced the risk of moderate visual loss, while scatter photocoagulation did not (6, 7). The high intensity of the laser burn used in the ETDRS protocol was associated with enlargement of laser scars, acute induction of choroidal neovascularization, long-term subretinal fibrosis, and frequent visual field loss (8). The Diabetic Retinopathy Clinical Research Network (8) and other researchers (9, 10) recently recommended mild grid laser photocoagulation.

Intravitreal anti-VEGF drugs have continuously shown good clinical effectiveness without significant undesirable side effects and achieved vision gains of ten or more letters on ETDRS charts in 50% of treated eyes (4). In addition, intravitreal anti-VEGF drugs can improve diabetic retinopathy but require repeated injections (1). Intravitreal corticosteroids are used when anti-VEGF agents fail, although there were complications such as ocular hypertension and formation of cataract. Despite the current availability of a number of treatments for DME, only a proportion of patients recover good vision (≥ 6/12 or 20/40) (4), and thus the search for newer therapies is in order.

This article outlines a new treatment protocol based on the theory that peripheral ischemia is the primer for angiogenesis with the responder site being the fovea.

## THE HYPOTHESIS/THEORY

Technique

1. Maximal pupil dilation (cyclopentolate 1%, mydriacyl 1%, phenylephrine 10%)

2. Minimal energy to reduce blanching panretinal photocoagulation laser burns (argon green, 0.1 second; 200 microns spot size; 1 place apart.)

3. Use of high-resolution wide-field laser lens (17) (Volk Optical, Mentor, Ohio, USA)

4. Treat regions from the outer periphery to equator with special attention to any ischemic areas apparent on ultrawide field intravenous fluorescein angiography (IVFA) (a minimum of 1000 and a maximum of around 2000 shots)

5. No need for retrobulbar anesthesia. Prepare patients with a low pain threshold by administering nonsteroidal anti-inflammatory medications an hour before the procedure

6. The laser is in a continuous mode with 0.1 sec pause and session lasting approximately five minutes.

Peripheral ischemia is an important finding in eyes with DME (11-14), which is highlighted even more by new technological advances in wide-angle fluorescein angiography. The modern approach suggests that treating this peripheral ischemia is a pivotal issue in DME therapy. Namely, peripheral ischemia leads to up-regulation of VEGF (14-16), and ablation of the periphery would result in down-regulation of VEGF. The use of recently available wide-angle lenses (17-19) in the application of the laser beam to extreme outer periphery has given fresh impetus to this new therapy. Furthermore, peripheral laser photocoagulation enhances formation of posterior vitreous detachment (PVD) (20), which enhances DME resolution. To prevent the immediate transient worsening of DME after laser, intravitreal anti-VEGF (with or without corticosteroid implant) is initially administered along with laser therapy. Multiple intravitreal injections like anti-VEGF agents increases the likelihood for PVD formation as well (21). Photocoagulation leads to increased oxygen supply to the remaining retina, especially the area of macula. This results in retinal vasoconstriction and a decrease in DME, avoiding the need for both focal therapies of the posterior pole and repeated anti-VEGF injections. In addition, it protects against the occurrence of neovascular glaucoma and vitreous hemorrhage.

EVALUATION OF THE HYPOTHESIS/IDEA

Half a decade ago, Wise and Wangvivat (22) thought that the macula mounts an exaggerated response to peripheral retinal disorders, such as diabetic retinopathy. Recently, Otani et al. (23) showed again that peripheral retinal vascular leakage could lead to serous retinal detachment in the macula. More recently, peripheral vascular leakage was documented in diabetic retinopathy by Oliver (12). The macula appears to act as a sink where pathologic processes occurring outside can secondarily evolve by the gravitational leakage, VEGF up-regulation, or vitreous biochemical changes leading to vitreomacular interface changes.

The ability to identify and hence treat ischemia of the peripheral retina may control DME since the nonperfused retina acts as a major source of VEGF production. The link between DME and peripheral retinal nonperfusion was initially described by Shimizu et al. in 1981 and in later years (24-26) using a 60o wide-angle IVFA camera. Additional findings of peripheral nonperfusion have been strongly correlated with DME (27). Recently, midperipheral nonperfusion was detected in 84% of eyes with DME and nonproliferative retinopathy (28). This was confirmed by other studies using ultrawide field imaging (15):

Peripheral vascular nonperfusion was detected in around two-thirds of eyes with severe non-proliferative diabetic retinopathy (non-PDR) and proliferative diabetic retinopathy (PDR) using ultrawide field IVFA. Angiographic characteristics of 264 eyes of 143 patients were evaluated. Among untreated eyes with nonproliferative retinopathy and DME, there was a trend for association of DME and peripheral nonperfusion (p = 0.065) (12, 13). According to Wessel et al. (29), eyes with retinal ischemia have a 3.75 times greater chance of developing DME.

Visualization of the peripheral retina is important for detailed angiographic retinal examination as well as for delivery of peripheral PRP in proliferative retinal diseases. Retinal ischemia is best visualized with IVFA. Traditional IVFA employs retinal photography that visualizes 30° of the retina at one time. The ETDRS developed the protocol of seven-standard fields (7SF) photographed areas and these are combined give close to 75° of visualization. With the advent of ultrawide-field fluorescein angiography (Optos 200Tx, Dunfermline, Scotland), 200° of retina is seen in a single photograph. Various contact and non-contact lens systems are available to allow panoramic visualization and therapy of the retina (19). The wide-field contact lens system (SuperQuad 160) uses even a wider aperture to expand the field of view, and similarly for the miniaturized HR wide-field contact lens (17, 18).

This newly described approach avoids focal therapy, sparing the posterior pole (and circumventing enlargement of macular scars with time) (30). Thus, repeated prolonged anti-VEGF therapy or dexamethasone implant are dodged since they (especially the latter) carry the risk of endophthalmitis, retinal vascular occlusion (31), and various potential systemic side effects. Wessel et al. (29) proposed combining focal macular laser, anti-VEGF pharmacotherapy and retinal photocoagulation to areas of ischemia. Takamura et al. (32) noted that targeted retinal photocoagulation for nonperfused areas decreased the risk of recurrence of DME after intravitreal injection of bevacizumab.

Our technique places laser therapy as the core treatment while others have used it as adjunctive to focal or anti-VEGF or combination. Although conventional laser PRP is somewhat effective in decreasing angiogenesis, it causes side effects, such as retinal scarring at the arcade and decreased peripheral, color, and night vision (33). Aiming at the extreme periphery and applying minimal laser energy can significantly decrease the above complications. It should be noted that patients do not notice many scotomas following conventional PRP because of perceptual compensational filling-in generated by a mechanism of plasticity of the visual cortex (34).

Several reports regarding light-energy PRP also referred to the technique as minimum intensity photocoagulation (MIP) (35, 36). Limited studies found that MIP is associated with fewer complications. In one study, MIP was associated with decrease in foveal thickness on OCT 12 months after therapy (36). 

Retinal photocoagulation produces its therapeutic benefit by the following mechanisms:

1. Destruction of the most metabolically active cells, decreasing the ischemic drive and secretion of angiogenic factors

2. Reduction of the total retinal oxygen demand as well as improvement of intraretinal oxygen delivery (37, 38)

3. Vasoconstrictive effect with the resultant decrease in vascular leakage (39, 40)

4. Facilitation of PVD formation (release of vitreomacular traction, increased oxygen in the vitreous cavity).

CONSEQUENCES OF THE HYPOTHESIS AND DISCUSSION

Traditional angiography is likely to miss major peripheral nonperfusions. Identification of specific areas of the nonperfused retina enables targeted extrafoveal laser photocoagulation in the treatment of DME. In the end, these areas of nonperfusion advance and necessitate serial wide-field angiograms and repeated selective peripheral laser. If additional laser treatment is required, it can be applied in a step-wise fashion. In practical terms, one session of panretinal peripheral laser is more convenient for the patient and clinician, especially in centers that lack wide-angle IVFA systems. Instead of repetitive VEGF suppression, we offer peripheral (preequatorial) PRP resulting in decreased VEGF production, stabilization or improvement in DME, and long-term protection from both PDR and neovascular glaucoma. This procedure is applicable for all patients with more laser treatments applied.

Anti-VEGF therapies, either corticosteroids or specific anti-VEGF agents, are given initially to act as a “jump” start and to remove the intraocular VEGF. As the outer-end periphery constantly produces new VEGF, new areas of nonperfusion in the posterior pole, additional injections may be needed at a reduced frequency. This approach reduces the cost of treatment substantially, as anti-VEGF injections are more than just a time-consuming burden for the health service. On the other hand, one session of laser is easily affordable, accessible, and attractive. The scheme currently employed for anti-VEGF application is scheduled as two injections in the first year and a single injection yearly vs. 12 injections in the study that lead to approval of the agent (4). We expect that there will be a need for anti-VEGF therapy as the outer-end periphery is not accessible to the most of up-to-date wide-angle laser lenses. These outer-end areas are accessible to peripheral cryopexy or indirect laser retinopexy with scleral indentation.

 Gardner et al (41) noted improvement in DME in poor vision eyes after panretinal photocoagulation (PRP). Their study group consisted of cases where PDR was managed using green laser (3-4 weekly sessions). Six months later, 13 of the 18 eyes demonstrated reduction of DME. The current technique is to be applied in proliferative and nonproliferative retinopathy, and the laser is to be administered preferentially to the outer-end periphery, unlike the ETDRS protocol (41). There is also benefit from initial use of anti-VEGF in combination with peripheral PRP. Ferraz et al. (42) in a randomized study compared PRP with intravitreal ranibizumab versus PRP alone in DME. Visual acuity was significantly better at 6 months in the ranibizumab group; central macular thickness decreased significantly at 6 months in the ranibizumab group and was unchanged in the photocoagulation group.

In conclusion, more than 25 years ago, the ETDRS proved that focal laser photocoagulation decreased the risk of moderate visual loss. The advent of anti-VEGF therapy and refinement of retinal imaging techniques have revolutionized our approach to DME. Ultra wide-field angiography provided visualization of peripheral retinal ischemia in DME, supporting the hypothesis that areas of untreated, retinal nonperfusion may be the sources of biochemical mediators that promote macular edema. Mild laser ablation to the outer-end periphery would theoretically decrease DME via several mechanisms downregulation of VEGF and other mediators, induction of PVD, decrease in retinal periphery leakage towards the fovea, and increase in the oxygen availability to the posterior pole. Research has begun to test this hypothesis in a clinical setting.
